# Supplementary material for: The Spurious Prospective Associations Model (SPAM): Explaining longitudinal associations due to statistical artifacts
Source: PLoS One. 2025 Sep 2;20(9):e0331609. doi: 10.1371/journal.pone.0331609 (PMC12404380; doi:10.1371/journal.pone.0331609)
Supplement: Attachment — Submitted filename: PLOS ONE Review SPAM.pdf [file pone.0331609.s001.pdf]

PLOS ONE Review

Title:- The Spurious Prospective Associations Model (SPAM): Explaining longitudinal associations due to statistical artifacts

Authors:- Kimmo Sorjonen, Ph.D., Karolinska Institutet, Solna, SWEDEN

Summary

This manuscript introduces the Spurious Prospective Associations Model (SPAM) as an alternative explanation for observed longitudinal associations between psychological constructs, challenging the interpretation of cross-lagged effects as evidence of true causal relationships. The authors provide a formalization of the model, apply it to several published meta-analyses, and demonstrate its ability to reproduce observed effects without assuming true prospective causality. The work is methodologically rigorous, addresses a significant issue in longitudinal data analysis, and is relevant to the readership of PLOS ONE.

This work makes a valuable contribution to the methodological literature in psychology and epidemiology by providing a robust alternative to conventional cross-lagged panel models. It is methodologically sound, theoretically well-motivated, clearly written, and adheres to PLOS ONE's scientific and ethical standards.

I recommend Minor Revisions before acceptance.

Comments to the Author

- ⇨ Technical Soundness & Data Support for Conclusions

The manuscript is technically sound, with robust methodology and transparent reporting. The conclusions are well-supported by the data, and the analytic approach is appropriate for the research question. The work meets high standards for technical rigor and data-driven inference)

- ⇨ Statistical Rigor
  - ✓ Assessment:-
    - Generally acceptable with minor clarifications needed
  - ✓ The manuscript demonstrates a high standard of statistical rigor. The analytic approach is methodologically sound, transparent, and well-justified for the research question. The results are robustly supported by appropriate statistical analyses, and the limitations are candidly addressed. The work meets the expectations for statistical rigor in high-quality empirical research

- ⇨ Methodological Standards
  - ✓ The manuscript meets or exceeds methodological standards for publication in PLOS ONE. The research design, analytic approach, transparency, and reporting are all exemplary. The work is a model of methodological rigor and transparency, and the conclusions are well-supported by the evidence presented. No major methodological concerns are identified.
- ⇨ Conclusion Support
  - ✓ The conclusions presented in the manuscript, "The Spurious Prospective Associations Model (SPAM) Explaining longitudinal associations due to statistical artifacts," are well-supported by the data, analyses, and arguments provided.
  - ✓ The authors clearly demonstrate, both conceptually and empirically, that many previously reported prospective associations in longitudinal psychological research can be explained by statistical artifacts specifically, regression to the mean, imperfect measurement reliability, and general associations between constructs rather than by true causal effects.
- ⇨ Data Repository Compliance
  - ✓ Open Data and Code
  - ✓ All relevant data, analytic scripts, and supporting materials are made available via the Open Science Framework, allowing for full transparency and reproducibility.
- ⇨ Presentation & Language
  - ✓ The manuscript is well organized and communicates complex statistical concepts effectively. The introduction, methods, and results are clearly delineated, and the use of figures and tables enhances comprehension. The writing is accessible to interdisciplinary researchers while maintaining technical precision.
- ⇨ Detailed Feedback
  - ✓ Strengths
    - Rigorous methodological approach
    - Transparent and reproducible analysis
    - Important implications for the interpretation of longitudinal data.
- ⇨ Major Issues

- ✓ After a thorough review of the manuscript The Spurious Prospective Associations Model (SPAM) Explaining longitudinal associations due to statistical artifacts (PONE-D-24-52718), I find the work to be methodologically rigorous and clearly presented.
- ✓ However, for a high-impact journal such as PLOS ONE, several major issues should be addressed to strengthen the manuscript and its implications for the field

⇨ Minor Issues

- ✓ The model does not account for measurement error via latent variables, which could strengthen its robustness
- ✓ Further comparison to models like the Random Intercept Cross-Lagged Panel Model (RI-CLPM) could enhance the discussion of alternatives

⇨ Suggestions

- ✓ Model Comparison:-
  - Consider including a brief discussion of how SPAM compares with other modern alternatives such as RI-CLPM or Latent Change Score models
- ✓ External Validation:-
  - While the simulations are compelling, a worked example using real raw data (not just correlations) could strengthen the argument
- ✓ Practical Implications:-
  - Consider providing examples of how SPAM might inform decisions in clinical, social, or policy interventions..

⇨ Comments to the Editor

- ✓ Competing Interests:-
  - The authors declare no competing interests.
- ✓ Funding:-
  - The authors received no specific funding for this work.
- ✓ Ethics:-
  - The study does not involve human or animal subjects. The ethics statement appropriately notes N/A. There are no identifiable ethical issues.
- ✓ Data Availability:-

- The data, analytic scripts, and supporting materials are fully available without restriction, in line with PLOS ONE’s data policy.

⇨ Recommendation

✓ Accept with minor revisions as suggested above
